# The relationship between poor nutritional status and progression of aortic calcification in patients on maintenance hemodialysis

**DOI:** 10.1186/s12882-018-0872-y

**Published:** 2018-03-20

**Authors:** Teppei Okamoto, Shingo Hatakeyama, Hirotake Kodama, Hirotaka Horiguchi, Yuka Kubota, Koichi Kido, Masaki Momota, Shogo Hosogoe, Yoshimi Tanaka, Tooru Takashima, Fumitada Saitoh, Tadashi Suzuki, Chikara Ohyama

**Affiliations:** 1 Department of Urology, Oyokyo Kidney Research Institute Aomori Hospital, 101-1 Okabe, Aomori, 038-0003 Japan; 2 Department of Urology, Hirosaki University Graduate School of Medicine, 5 Zaifu-chou, Hirosaki, 036-8562 Japan; 3 Department of Urology, Oyokyo Kidney Research Institute, Hirosaki, Japan; 4 Department of Advanced Transplant and Regenerative Medicine, Hirosaki University Graduate School of Medicine, Hirosaki, Japan

**Keywords:** Malnutrition, Geriatric nutritional risk index, Aortic calcification progression, Hemodialysis

## Abstract

**Background:**

Although aortic calcification has a significant negative impact on prognosis in patients on hemodialysis (HD), risk factors for aortic calcification progression remain unclear. The aim of this study was to investigate the relationship between malnutrition and aortic calcification progression in patients on HD.

**Methods:**

Between April 2015 and October 2016, we treated 232 patients on HD. Of those, we retrospectively evaluated data from 184 patients who had had regular blood tests and computed tomography (CT) scans. The abdominal aortic calcification index (ACI) was quantitatively measured by abdominal CT. Nutritional status was evaluated using the Geriatric Nutritional Risk Index (GNRI). A normalized treatment ratio of functional urea clearance was evaluated by Kt/V. The difference in ACI values between 2015 and 2016 was evaluated as a ΔACI, and patients were stratified into two groups according to ΔACI value: high (≥75th percentile, ΔACI-high group) and low (<75th percentile, ΔACI-low group). Variables such as age, sex, comorbidities, dialysis vintage, serum data, and GNRI were compared between ΔACI-high and ΔACI-low patients. Factors independently associated with a higher ΔACI progression (ΔACI ≥75th percentile) were determined using multivariate logistic analysis.

**Results:**

Median values of ACIs in 2015 and 2016 were 40.8 and 44.6%, respectively. Of 184 patients, 125 (68%) patients experienced ACI progression for 1 year. The median ΔACI and 75th percentile of ΔACI were 2.5% and 5.8%, respectively. The number of patients in the ΔACI-low and ΔACI-high groups were 128 (70%) and 56 (30%), respectively. There were significant differences in sex, presence of diabetic nephropathy, HD vintage, serum albumin, serum phosphate, C-reactive protein, intact parathyroid hormone, Kt/V, and GNRI. Multivariate logistic regression analysis revealed that independent factors associated with a higher ΔACI progression were male sex, serum phosphate levels, HD vintage, and GNRI of < 90.

**Conclusions:**

Our results suggest that poor nutritional status is an independent risk factor for the progression of aortic calcification. Nutrition management may have the potential to improve progression of aortic calcification in patients on HD.

**Trial registration:**

UMIN Clinical Trials Registry UMIN000028050.

## Background

Arterial calcification is a typical phenomenon of patients with chronic kidney disease (CKD) and those undergoing hemodialysis (HD) [1, 2]. Arterial calcification is strongly related to all-cause and cardiovascular mortality and morbidity in patients with CKD [3, 4]. Both classical and non-classical risk factors have been implicated in vascular calcification progression among patients undergoing HD. Classical risk factors which can predict coronary heart disease outcomes are male sex, hypertension, smoking, and diabetes mellitus [5]. Non-classical risk factors (uremia-related factors) such as serum phosphate, calcium phosphate product, and intact parathyroid hormone (i-PTH) were significantly related to arterial calcification in HD patients [6–8]. There are few promising treatments to decrease arterial calcification [9]. Therefore, prevention is crucial to reduce the mortality and morbidity of patients on maintenance HD.

Malnutrition is highly prevalent among patients on maintenance HD. Malnutrition is significantly associated with arterial sclerosis, cardiovascular disease (CVD), and total mortality of HD patients [10]. There exist several tools to evaluate malnutrition. Of these, the Geriatric Nutrition Risk Index (GNRI), calculated using serum albumin, height, and dry weight, is a simple and accurate nutritional indicator for patients on maintenance HD [11]. Several reports have suggested that GNRI is a significant predictor of mortality from CVD [10]. Furthermore, a recent study reported that GNRI was significantly related to the severity of aortic calcification in CKD patients not on HD [12]. However, the relationship between malnutrition and progression of vascular calcification remains unclear. Here we aimed to investigate the relationship between progression of aortic calcification and malnutrition in patients on maintenance HD.

## Methods

### Study design

This was a retrospective, single center, observational study. The study was conducted in accordance with the ethical standards of the Declaration of Helsinki and was approved by the Ethics Committee of Hirosaki University Graduate School of Medicine (authorization number 2016–225). The participants in this study provided their verbal informed consent, and it was recorded in medical chart. Pursuant to the provisions of the ethics committee and ethics guidelines in Japan, written consent was not required in exchange for public disclosure of study information in the case of retrospective and/or observational study using materials such as existing documentation. The ethics committees in Hirosaki University School of Medicine approved this consent procedure. The study information was open for public referral at http://www.med.hirosaki-u.ac.jp/~uro/html/IRB/IRBdoc.html.

### Patient selection

**Fig. 1:**
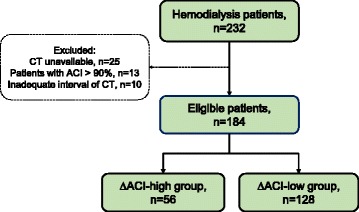
Patient selection and classification. We treated 232 hemodialysis (HD) patients who underwent 3–4 h-hemodialysis sessions 3 times/week. Of these, we excluded 25 patients with abdominal computed tomography (CT) unavailable. We excluded patients with severe AC (ACI > 90%) in 2015, with an inadequate interval (≤ 10 months) of abdominal CT scans. Finally, the remaining 184 HD patients were included. We divided patients into two groups according to the 75th percentile of ΔACI: ΔACI-high (ΔACI ≥5.8%) or ΔACI-low (ΔACI < 5.8%)

Between April 2015 and October 2016, we treated 232 patients who had undergone 3–4 h of maintenance HD or online hemodiafiltration using a dialysate containing 3.0 mEq/L calcium three times a week at the Oyokyo Kidney Research Institute in Aomori, Japan. Almost all patients had undergone annual abdominal computed tomography (CT) scans to detect incidental renal tumor and other malignancies. Of these, we excluded patients with severe aortic calcification, including calcification extending along the entire length and almost all of the circumference of the abdominal aorta, or who had an inadequate interval (≤10 months) of abdominal CT scans. Finally, we selected 184 patients who had undergone adequate abdominal CT scans (Fig. 1).

### Evaluation of outcome variables

Diabetic nephropathy (DMN) was defined as a cause of CKD. Current smoking was defined as smoking at least one cigarette during the study period. Previous history of CVD was defined as having any previous description of ischemic heart disease, cerebrovascular accident, or peripheral arterial disease recorded in the patients’ medical records. Every two months, patients underwent a routine laboratory exam before each HD session. We obtained laboratory data for serum phosphate, serum albumin, serum calcium, C-reactive protein (CRP), and i-PTH between April 2015 and October 2016. We used the mean blood pressure of six dialysis sessions on the day of blood and serum test as a representative value of blood pressure. Hypertension (HTN) was defined as having a systolic blood pressure of ≥140 and/or a diastolic blood pressure of ≥90 mmHg. Serum calcium level was corrected using the following formula: corrected calcium = total calcium + 0.8 × (4–serum albumin), if serum albumin level was < 4.0 g/dL. The normalized dialysis dose (Kt/V) was calculated using the following equation: Kt/V = −Ln (Ct/Co − 0.008 × t) + (4–3.5 × Ct/Co) × ΔBW/BW. Ct divided by Co represents the ratio of post-dialysis to pre-dialysis serum urea nitrogen, t represents dialysis time, and W (kg) represents post-dialysis body weight.

### Abdominal aortic calcification index (ACI)

We used CT scans (SOMATOM Perspective, Siemens Healthcare, Tokyo, Japan) to evaluate abdominal aortic calcification in all subjects. Images were obtained with a 5-mm slice thickness. Abdominal aortic calcification was semi-quantitatively measured from CT images of the area above the common iliac artery bifurcation by conducting 10 scans at 5-mm intervals, as described elsewhere [13]. Measurement of abdominal aortic calcification in 2015 and 2016 was performed simultaneously. Abdominal aortic calcification index (ACI, %) represents the calcification proportion in 12 sectors. ACI was calculated using following formula: ACI = (total score for calcification on all slices)/12/10 × 100 (%). We defined ACI values of > 90% as severe aortic calcification. The difference in ACI values between 2015 and 2016 (ΔACI) was calculated by subtracting the ACI value in 2016 (ACI-2016) from that in 2015 (ACI-2015). All procedures were conducted by a single physician before collecting patients’ clinical data and background information. We evaluated median ACI-2015, ACI-2016, ΔACI, and 75th percentiles of ΔACI. Patients were divided into ≥75th percentile of ΔACI (ΔACI-high group) and < 75th percentile of ΔACI (ΔACI-low group).

### GNRI

The GNRI is calculated by the following formula: GNRI = 14.89 × serum albumin (g/dL) + 41.7 × (body weight/ideal body weight). Ideal body weight was calculated from height and using a body mass index (kg/m^2^) of 22. We used mean GNRI between April 2015 and October 2016 as a representative value.

### Comparison

Variables such as age, sex, comorbidities, dialysis vintage, serum data, and GNRI were compared between patients in the ΔACI-high and ΔACI-low groups. Factors independently associated with a higher ΔACI progression (ΔACI ≥75th percentile) were determined using multivariate logistic regression analysis.

### Statistical analysis

Statistical analyses were conducted using SPSS version 22.0 (IBM Corporation, Armonk, NY, USA). Categorical variables (such as sex) are presented as percentages. Continuous variables with a normal distribution and are expressed as the mean (standard deviation; SD); those with a non-normal distribution are expressed as the median (interquartile range; IQR). Wilcoxon signed-rank test was performed to compare ACI-2015 and ACI-2016 values. Sex (0 = female, 1 = male), HTN (0 = absence, 1 = presence), DMN (0 = other, 1 = presence), current smoking (0 = absence, 1 = presence), and GNRI < 90 (0 = absence, 1 = presence) were included as binary variables in the model. Comparisons between ΔACI-high and ΔACI-low groups were performed using Fisher’s exact test or Chi-square test, Student’s *t*-test (normally distributed data), and Mann–Whitney *U*-test (non-normally distributed data). Multivariate logistic regression analysis was conducted to evaluate an independent predictor for higher ΔACI progression (ΔACI ≥75th percentile). Based on previous studies, well-known aortic calcification progression factors such as HD vintage, HTN, i-PTH, serum phosphate, corrected calcium level, DMN, sex, age, and current smoking were included in multivariable models. Odds ratios (ORs) with 95% confidence intervals (CIs) associated with each factor were calculated after adjusting for potentially confounding factors. Probability (*P*) values < 0.05 were considered statistically significant.

## Results

### Patient classification

**Fig. 2:**
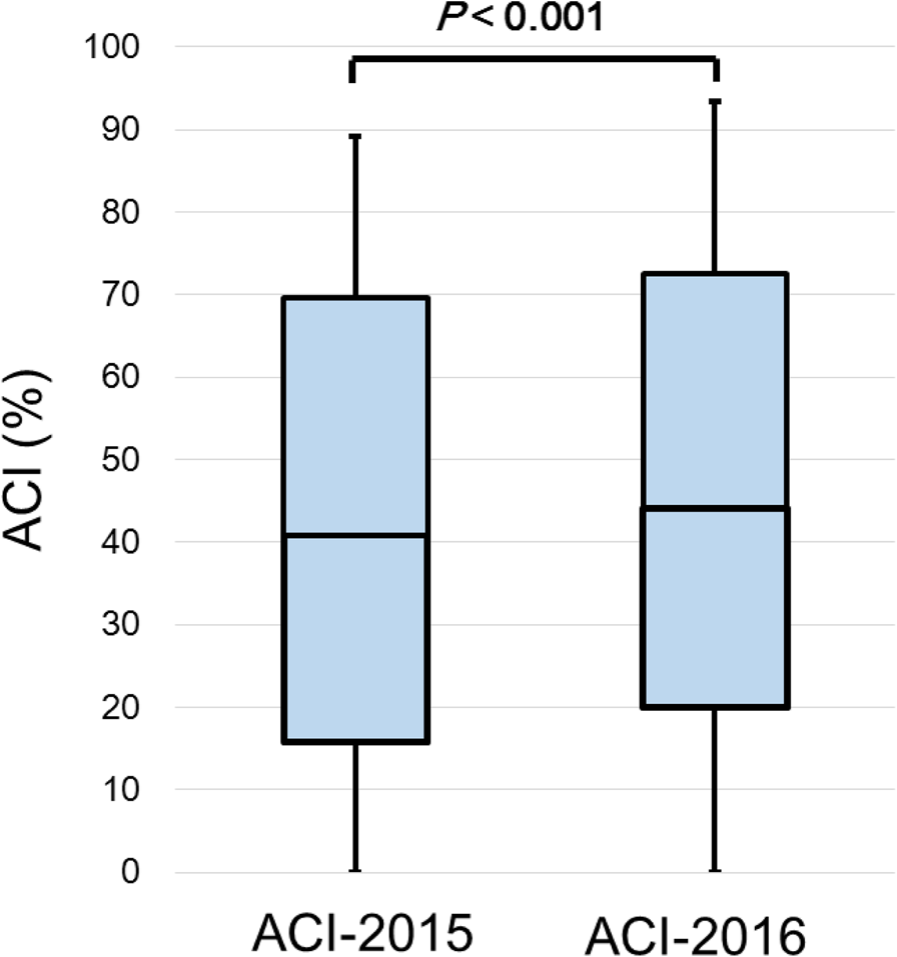
Wilcoxon signed-rank test between ACI-2015 and ACI-2016. Median ACI-2015 and ACI-2016 were 40.8% [interquartile range (IQR), 15.8–70.2] and 44.6% (IQR, 20.0–72.5), respectively. The ACI value in 2016 was significantly increased compared to that in 2015 (*P* < 0.001)

This retrospective study included 184 patients (108 males, 76 females) undergoing HD (Fig. 1). The median age was 66 [interquartile range (IQR), 58–76] years. The mean follow-up period was 11.8 [standard deviation (SD), 0.5] months. Median ACI-2015 and ACI-2016 were 40.8% (IQR, 15.8–70.2) and 44.6% (IQR, 20.0–72.5), respectively. Of the 184 patients, 125 (68%) experienced aortic calcification progression for 1 year. The ACI value in 2016 was significantly increased compared to that in 2015 (*P* < 0.001) (Fig. 2). Median ΔACI and 75th percentiles of ΔACI were 2.5% and 5.8%, respectively. Numbers of patients in the ΔACI-high and ΔACI-low groups were 56 (30%) and 128 (70%), respectively.

### Comparison of clinical characteristics between patients in ΔACI-high and ΔACI -low groups

**Fig. 3:**
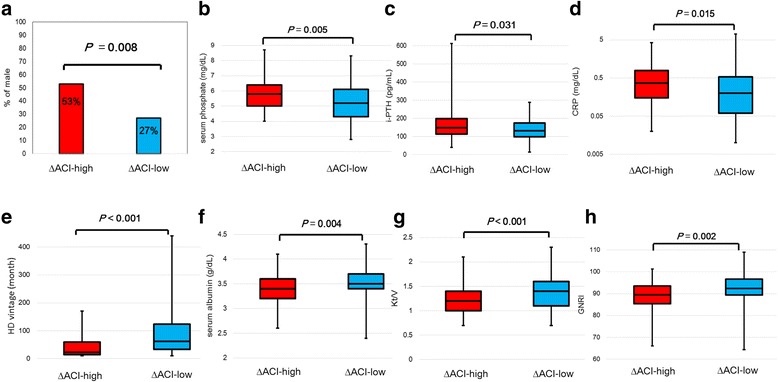
Comparison of clinical characteristics between the ΔACI-high and ΔACI-low groups. In the ΔACI-high group (ΔACI ≥5.8%), the proportion of males was significantly higher compared with the ΔACI-low group (ΔACI < 5.8%) (**a**). Among patients in the ΔACI-high group, serum phosphate (**b**), intact parathyroid hormone (i-PTH) (**c**), and CRP (**d**) were significantly higher compared with patients in the ΔACI-low group. Hemodialysis (HD) vintage (**e**), serum albumin (**f**), Kt/V (**g**), and Geriatric Nutritional Risk Index (GNRI) (**h**) were significantly shorter or lower in the ΔACI-high group than in the ΔACI-low group

**Fig. 4:**
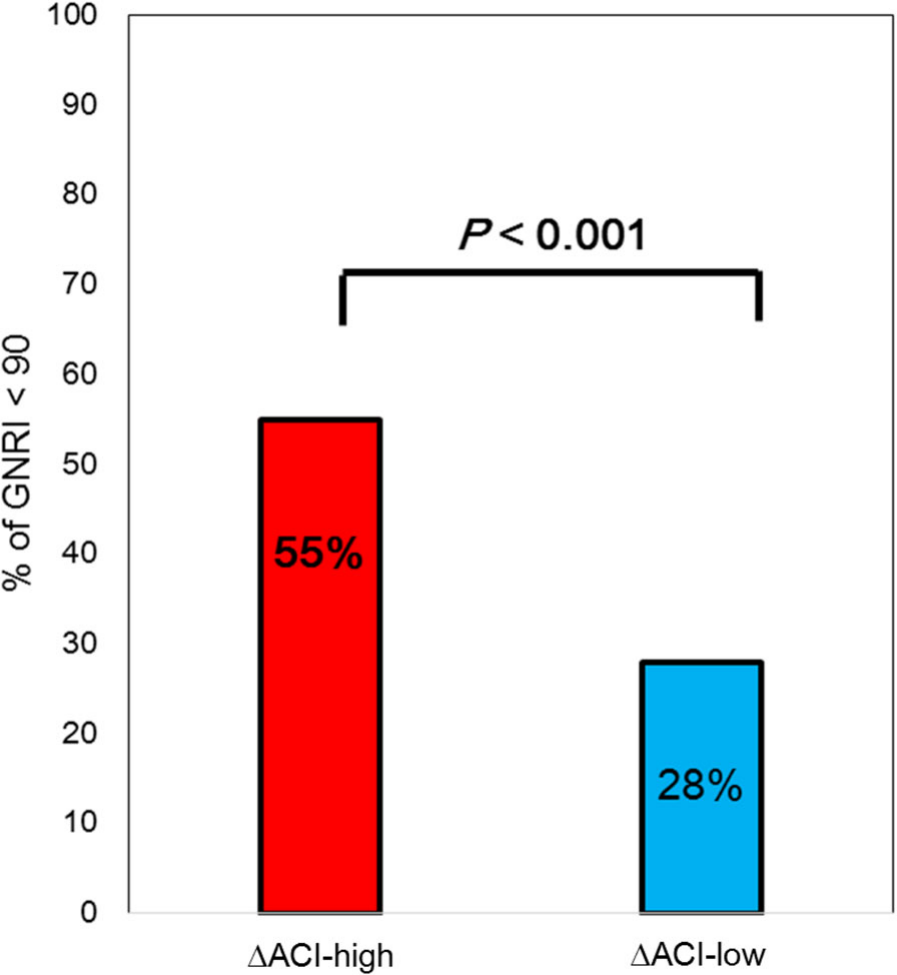
Comparison of the proportion of GNRI < 90 between patients in the ΔACI -high and ΔACI-low groups. In the ΔACI-high group (ΔACI ≥5.8%), the proportion of Geriatric Nutritional Risk Index (GNRI) < 90 was significantly higher compared with that in the ΔACI-low group (ΔACI < 5.8%)

The clinical characteristics and laboratory data of patients in the ΔACI-high and ΔACI-low groups are displayed in Table 1. There were no significant differences in age, prevalence of HTN, or current smoking, or corrected calcium level between the two groups. The proportions of male patients and patients with DMN were significantly higher in the ΔACI-high group than in the ΔACI-low group (Fig. 3a). Patients in the ΔACI-high group had significantly higher levels of serum phosphate (*P* = 0.005, Fig. 3b), i-PTH (*P* = 0.031, Fig. 3c), and CRP (*P* = 0.015, Fig. 3d) than those in the ΔACI-low group. In addition, patients in the ΔACI-high group had significant shorter HD vintage (*P* < 0.001, Fig. 3e), lower serum albumin level (*P* = 0.004, Fig. 3f), lower Kt/V (*P* < 0.001, Fig. 3g), and lower GNRI (*P* = 0.002, Fig. 3h) than those in the ΔACI-low group. The proportion of patients with poor nutritional status (GNRI < 90) was significantly higher in the ΔACI-high group than that in the ΔACI-low group (55% vs. 28%, *P* < 0.001) (Fig. 4).

**Table 1:** Clinical characteristic of the ΔACI-high and the ΔACI-low groups

	ΔACI-high	ΔACI-low	*P*-value
Number	56 (30%)	128 (70%)	–
Age^a^ (year)	68 (58–77)	65 (58–74)	0.149
Sex, male^b^, n	67 (53%)	41(27%)	0.008
Cause of CKD			
DMN^b^ (presence), n	31 (55%)	50 (40%)	0.04
Chronic glomerulonephritis^b^ (presence), n	10 (18%)	27 (21%)	0.692
Autosomal dominant polycystic kidney disease^b^ (presence), n	3 (5.0%)	5 (4.0%)	0.175
Others^b^ (presence), n	12 (21%)	46 (36%)	0.06
Modality of hemodialysis^b^			1.00
HD, n	49 (88%)	111 (87%)	–
Online hemodiafiltration, n	7 (12%)	17 (13%)	–
Systolic blood pressure^a^ (mmHg)	150 (133–161)	152 (141–167)	0.277
Diastolic blood pressure^a^ (mmHg)	78 (69–91)	78 (72–86)	0.776
HTN^b^ (presence), n	34 (61%)	97 (76%)	0.05
Current smoking^b^ (presence)	10 (18%)	16 (14%)	0.337
Previous history of CVD^b^ (presence), n	14 (25%)	29 (23%)	0.710
HD^a^ vintage (months)	22.5 (14.0–60.0)	62.0 (34.0–123)	< 0.001
Serum albumin^a^ (g/dL)	3.4 (3.2–3.6)	3.5 (3.5–3.6)	0.004
CRP^a^ (mg/dL)	0.36 (0.15–0.78)	0.20 (0.06–0.53)	0.015
GNRI^a^	89.5 (85.4–93.5)	92.3 (89.3–96.3)	0.002
GNRI^b^ < 90, n	31 (55%)	36 (28%)	< 0.001
Kt/V^a^	1.2 (1.0–1.4)	1.4 (1.1–1.6)	< 0.001
Serum phosphate^a^ (mg/dL)	5.8 (5.0–6.4)	5.3 (4.3–6.1)	0.005
Corrected calcium^a^ (mg/dL)	9.2 (8.8–9.5)	9.3 (8.9–9.7)	0.126
i-PTH^a^ (pg/mL)	149 (113–198)	131(98–174)	0.031

Comparison values are median (interquartile range; IQR)

*ACI* abdominal aortic calcification index, *CKD* chronic kidney disease, *DMN* diabetic nephropathy, *HD* hemodialysis, *HTN* hypertension, *CVD* cardiovascular disease, *CRP* C-reactive protein, *GNRI* Geriatric Nutrition Risk Index, *i-PTH* intact parathyroid hormone

^a^ Mann–Whitney U-test

^b^ Fisher’s exact test

### Independent risk factors for development of high ΔACI

**Fig. 5:**
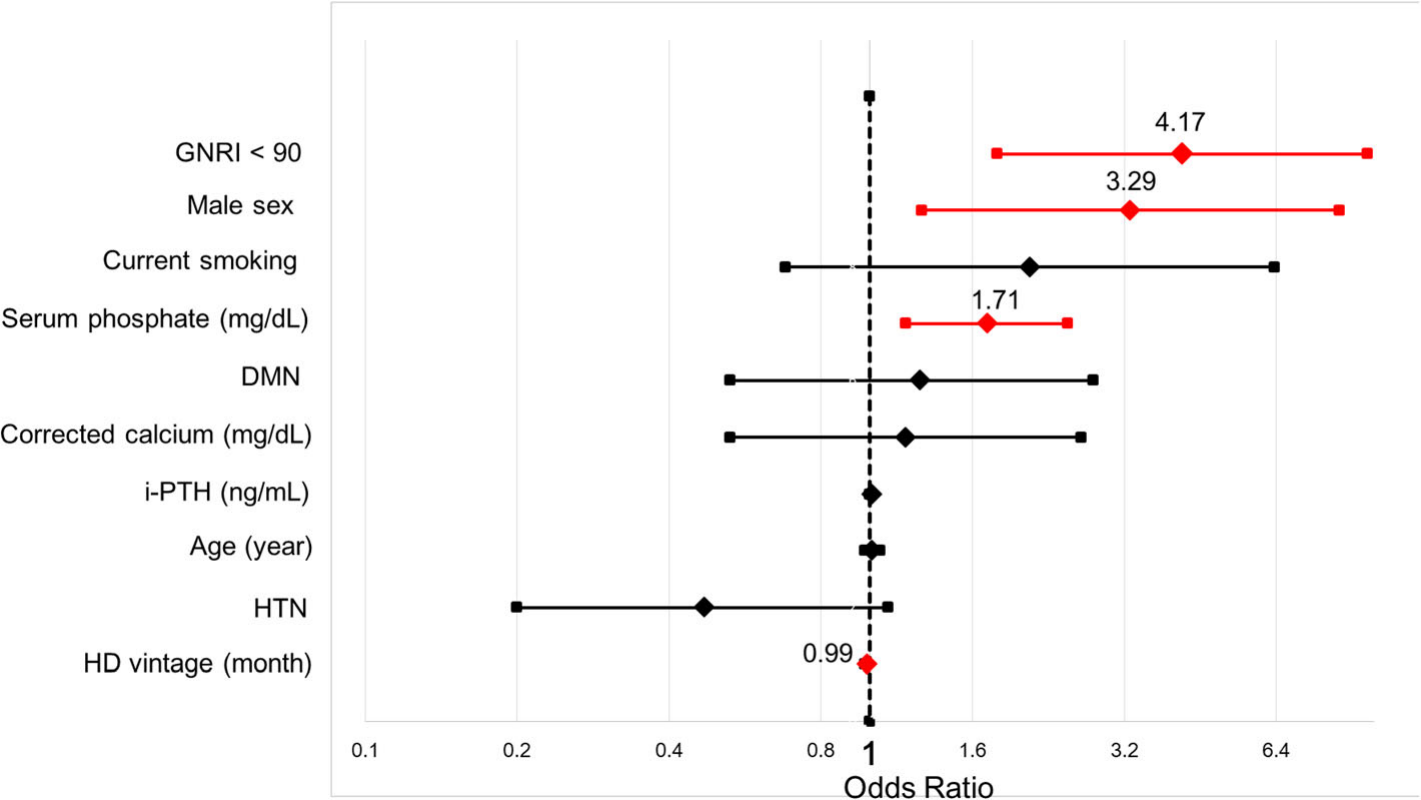
Factors associated with ACI progression. Independent risk factors for high ΔACI were evaluated using multivariate logistic regression analysis. Geriatric Nutritional Risk Index (GNRI) < 90, male sex, serum phosphate, and hemodialysis (HD) vintage were selected as independent risk factors for ACI progression

Independent risk factors for an ACI progression rate greater than the 75th percentile were evaluated by multivariate logistic regression analysis (Table 2). GNRI < 90 (OR = 4.17; 95% CI = 1.79–9.71), male sex (OR = 3.29; 95% CI = 1.27–8.53), serum phosphate (OR = 1.71; 95% CI = 1.18–2.47), and HD vintage (OR = 0.99; 95% CI = 0.98–0.99) were selected as independent risk factors for an ACI progression rate greater than the 75th percentile, after accounting for confounders of aortic calcification progression such as age, DMN, current smoking, HTN, i-PTH, and corrected calcium level (Fig. 5).

**Table 2:** Independent risk factors for high ΔACI by multivariate logistic regression analysis

Variable	Risk factor	*P-value*	Odds ratio	95% CI
GNRI	< 90	*0.001*	4.17	1.79–9.71
Sex	Male	*0.014*	3.29	1.27–8.53
Current smoking	Positive	*0.197*	2.08	0.68–6.35
Serum phosphate (mg/dL)	Continuous	*0.004*	1.71	1.18–2.47
DMN	Positive	*0.645*	1.26	0.53–2.78
corrected calcium (mg/dL)	Continuous	*0.694*	1.18	0.53–2.63
i-PTH (ng/mL)	Continuous	*0.055*	1.01	1.00–1.01
Age (year)	Continuous	*0.472*	1.01	0.98–1.05
HTN	Positive	*0.078*	0.47	0.20–1.09
HD vintage (month)	Continuous	*0.007*	0.99	0.98–0.99

*GNRI* Geriatric Nutrition Risk Index, *DMN* Diabetic nephropathy, *i-PTH* intact parathyroid hormone, *HTN* Hypertension, *HD* hemodialysis

## Discussion

In the present study, we investigated clinical parameters that were associated with aortic calcification progression, and found that male sex, serum phosphate level, HD vintage, and GNRI < 90 were significant factors for aortic calcification progression in patients on maintenance HD. The key finding was that poor nutritional status was a critical factor for progression of aortic calcification. To the best of our knowledge, this is the first report to demonstrate the relationship between nutritional status and aortic calcification progression in patients on maintenance HD.

Malnutrition is one of the most common complications in patients on maintenance HD and is associated with adverse clinical outcomes in these patients [10, 12]. Many factors can affect the nutritional and metabolic status of these patients, including protein energy wasting, catabolic effects of renal replacement therapy, metabolic and hormonal disorders, and comorbidities. Several tools have been designed to evaluate nutritional status. The Subjective Global Assessment (SGA) and Malnutrition-Inflammation Score (MIS) are indicators of malnutrition for patients on maintenance HD [14, 15]. A previous study demonstrated that using modified quantitative SGA, malnutrition was found to be the most important factor associated with the amount of calcified depositions in the radial arteries among patients on maintenance HD [16]. However, the SGA and MIS require subjective assessment by the examiner, and compatibility between evaluations of different examiners is difficult to achieve. On the other hand, the GNRI is a simple tool that requires only serum albumin, height, and dry weight assessment. Several studies have reported that GNRI is the simplest and most accurate indicator of nutritional risk in patients on HD [11], and that it is a significant predictor of mortality in patients with CKD [17–20]. One previous study suggested that the optimal cutoff value of GNRI for mortality of HD patients was 90, based on the highest positive likelihood and risk ratios for overall survival [17]. Moreover, a recent observational study revealed that lower GNRI was significantly associated with severe vascular calcification in patients with CKD not on HD [12]. Indeed, the present study demonstrated that patients with rapid ACI progression had significantly lower GNRI than those with slow ACI progression (89.5 vs. 92.3). Furthermore, our results suggested that HD patients with GNRI < 90 had an approximately 4-fold higher risk of rapid aortic calcification progression than those with GNRI ≥90. In sum, our finding was consistent with previous reports, and suggested that poor nutritional status was closely associated with progression of arterial calcification in patients on maintenance HD.

Although the precise mechanism and relationship between progression of aortic calcification and malnutrition has not been fully elucidated, one possible explanation for the progression of aortic calcification is its association with malnutrition-inflammation-atherosclerosis (MIA) syndrome and/or fetuin-A. CKD-related inflammation causes malnutrition and progressive atherosclerosis, which is known as MIA syndrome [21]. Fetuin-A, a circulating calcium-regulatory glycoprotein, was reported to be closely associated with MIA syndrome [22]. Fetuin-A inhibits vascular calcification in patients with CKD by removing calciprotein particles (calcium phosphate-containing nano-aggregates) [23]. Both the activity and serum levels of fetuin-A are decreased in dialyzed patients [24]. Furthermore, previous reports have shown that fetuin-A levels were positively correlated with serum albumin, serum prealbumin, and SGA in dialyzed patients [25–27]. Our results regarding the relationship between malnutrition and rapid aortic calcification progression may support these findings. However, few studies have provided sufficient evidence of the effectiveness of nutritional management to increase fetuin-A levels and decrease progression of vascular calcification. Further prospective studies will be needed to determine that nutrition treatment for HD patients can prevent progression of vascular calcification.

Several studies have reported that patients on maintenance HD experience more rapid progression of vascular calcification than do healthy individuals [28]. Our results suggested that classical and non-classical risk factors had a significant impact on aortic calcification progression. These findings were consistent with previous studies which demonstrated that male sex [29, 30], hyperphosphatemia [6–8] accelerate the process of vascular calcification and atherosclerosis among patients with CKD and/or who are on maintenance HD. On the other hand, the relationship between HD vintage and progression of vascular calcification has been controversial. Previous studies have revealed that dialysis vintage was positively correlated with vascular calcification progression [29, 31]. However, another study demonstrated the converse relationship [32]. In the present study, patients with rapid progression of ACI had significantly shorter HD vintage than those with slow progression (22.5 vs 62.0 months). A previous study demonstrated that the initiation of hemodialysis triggered apoptosis of vascular smooth muscle cell, which induced rapid and extreme vascular calcification [33]. This finding may imply that rapid progression of vascular calcification occurred in the early transition period following initiation of HD. Our previous study revealed that even after renal transplantation, many patients who experienced maintenance HD showed significant progression of aortic calcification for 10 years [34]. This finding may imply that the progression of vascular calcification was significantly enhanced by the presence of CKD. Moreover, progression of vascular calcification is closely related with poor all-cause and cardiovascular mortality-free survival rates [35, 36]. Because there is a complex web of interactions between the progression of vascular calcification and several risk factors, the mechanism of vascular calcification remains unclear.

Several limitations of this study should be described. First, this study was conducted retrospectively at a single center. In addition, its small sample size and selection biases prevent us from obtaining definitive conclusions. Second, our semi-quantitative measurement of aortic calcification did not enable us to evaluate its change in thickness. Third, we could not address the impact of medications such as phosphate binders, cinacalcet, and antihypertensive drugs, nor of dietary habits and normalized protein catabolic rates, which represent protein intake. Finally, because the GNRI requires only serum albumin, body weight, and height, it does not reflect total body composition, which consists of muscle mass, fat, and total body water. Despite these limitations, using a simple and accurate nutritional assessment tool, we were able to demonstrate an independent association between malnutrition and progression of aortic calcification in patients on maintenance HD. In our next study, we plan to address the relationship between malnutrition, fetuin-A, and aortic calcification.

## Conclusions

A potential role between malnutrition and aortic calcification may exist in patients on maintenance HD. Our findings may encourage clinicians to pay greater attention to nutrition management to prevent the progression of arterial calcification.

## Abbreviations

ACI: Abdominal aortic calcification index
CKD: Chronic kidney disease
CRP: C-reactive protein
CT: Computed tomography
CVD: Cardiovascular disease
DMN: Diabetic nephropathy
GNRI: Geriatric nutritional risk index
HD: Hemodialysis
HTN: Hypertension
i-PTH: Intact parathyroid hormone
MIA: Malnutrition-inflammation-atherosclerosis
MIS: Malnutrition-inflammation score
SGA: Subjective global assessment
